# Diagnostic and prognostic value of adipose tissue content and distribution indicators for normal weight obesity in young women

**DOI:** 10.1038/s41598-025-12262-6

**Published:** 2025-09-30

**Authors:** Waldemar Pluta, Anna Lubkowska, Wioleta Dudzińska

**Affiliations:** 1 Department of Functional Diagnostics and Physical Medicine, Pomeranian Medical University in Szczecin, Żołnierska 54, 71-210 Szczecin, Poland; 2 Institute of Biology, University of Szczecin, Felczaka 3C, 71-412 Szczecin, Poland

**Keywords:** Normal weight obesity, Cut-off point, Dual-energy x-ray absorptiometry, Fat distribution, A/G ratio, Diagnostic markers, Endocrine system and metabolic diseases, Metabolic disorders

## Abstract

The rise in global obesity and related health risks has highlighted the importance of precise body composition analysis, especially in individuals who appear healthy according to Body Mass Index (BMI). This study examines the diagnostic and prognostic significance of body composition indicators for assessing cardiometabolic risk in young women with normal weight obesity (NWO). The research included 330 women aged 18–24 years from northwestern Poland with normal BMI (18.5–24.9 kg/m^2^), using dual-energy X-ray absorptiometry (DXA) for detailed body composition assessment. A key achievement of the study was establishing a new cut-off point for body fat percentage (PBF) at 35.78%, which effectively identified individuals at increased cardiometabolic risk. Results showed that 27.3% of participants were classified as having NWO and exhibited higher insulin levels, increased HOMA-IR scores, and a lipid profile indicative of greater cardiometabolic risk, including elevated total cholesterol, LDL-C, non-HDL-C, and triglycerides, alongside reduced HDL-C levels. The android-to-gynoid (A/G) fat ratio emerged as a significant predictor, correlating positively with insulin resistance and negatively with HDL-C levels. These findings highlight the limitations of BMI and underscore the need for comprehensive body composition analysis. Identifying the NWO phenotype early could prompt preventive lifestyle interventions, even in those with a normal BMI.

**Supplementary Information:**

The online version contains supplementary material available at 10.1038/s41598-025-12262-6.

## Introduction

The number of people with obesity and its accompanying health consequences is growing every year. According to a 2022 report by the NCD Risk Factor Collaboration, over 1 billion people in the world live with obesity. This global trend is reflected in national statistics, with Poland showing particularly concerning trends among women—4.8 million Polish women suffer from obesity (which is 24.6% of all Polish women). This is an increase of 7.4 percentage points since 1990. In this respect, Poland ranks 108th in the global ranking in terms of the incidence of obesity in women^1^. The development of obesity is influenced by both lifestyle factors (e.g. low physical activity, poor diet) as well as genetic and epigenetic factors. Excess body fat can affect the entire body, including the cardiovascular, endocrine, gastrointestinal, and central nervous systems^2^. This rapid increase in obesity prevalence underscores the necessity for a comprehensive grasp of the processes underlying the development of obesity and the implementation of efficacious diagnostic techniques.

Research indicates that the period between 18 and 25 years of age represents a critical window for the development of overweight and obesity^3^. This developmental stage, marking the transition from adolescence to adulthood, is characterized by increasing autonomy in lifestyle choices and significant changes in social and environmental influences. During this period, young adults often experience their first independent living situation, begin higher education or enter the workforce, and establish long-term dietary and physical activity patterns^4^. This period is characterized by significant lifestyle changes, including a reduction in physical activity, irregular eating patterns and increased stress levels^5^. These changes can contribute to unfavorable alterations in body composition. Studies demonstrate that habits formed during this transition period often persist into later adulthood, making it a crucial point of intervention for the prevention of obesity-related disorders^6^.

Gender is also an important aspect influencing obesity and body composition. Women and men differ in adipose tissue’s amount, distribution and metabolic function. The physiological range of body fat content in women is 25–31%, while in men it is only 18–24%. In women, fat tissue accumulates mainly in the thighs and buttocks (gynoid distribution), while in men, fat tissue accumulates in the abdominal cavity (android distribution). Women have greater subcutaneous fat stores, while men are more likely to store visceral fat. These differences are primarily driven by sex hormones, particularly estrogen and testosterone, which regulate adipogenesis and fat distribution patterns. Due to the significant physiological differences between the sexes in adipose tissue metabolism, hormonal regulation, and cardiovascular risk profiles, this study focused exclusively on young women to ensure rigorous methodology and eliminate sex-related confounding variables^7–13^.

The standard diagnostic tool for assessing excess body weight is the body mass index (BMI) (with established categories: underweight < 18.5 kg/m^2^, normal weight 18.5–24.9 kg/m^2^, overweight 25–29.9 kg/m^2^, and obesity ≥ 30 kg/m^2^), which, however, has many limitations. BMI does not distinguish between fat and muscle mass, which means that it does not always reflect the actual metabolic state of a person^14^. Studies have shown that people with a normal BMI may have high levels of body fat, which is associated with an increased risk of cardiometabolic disorders such as insulin resistance, dyslipidemia and hypertension^15–17^. This condition, termed "normal weight obesity" (NWO)—defined as having a normal BMI (18.5–24.9 kg/m^2^) but excess body fat percentage, is becoming increasingly important in epidemiological and clinical studies. The incidence of NWO depends on the population, ranging from ~ 29%^18^ to 46%^19^. According to data from 2015, the prevalence of NWO worldwide is estimated at about 30%^20^. Population studies conducted in Europe have shown that NWO occurs more often in women than in men^21,22^. Although BMI is a simple and widely used indicator of obesity, underestimation of obesity based on BMI criteria may lead to “masking” of cardiometabolic risk, delayed diagnosis, prevention and appropriate treatment^23^. This highlights the need to use more accurate methods of assessing health risks than BMI^17,23–25^.

A key indicator in the diagnosis of NWO is the percentage of body fat (PBF), which provides a more accurate assessment of metabolic health risk than BMI alone. Importantly, there is no consensus on the body fat percentage thresholds^19,26–28^ with cutoff values ranging from 19%^29^ to 32.6%^26^ for men and 29.2%^30^ to 44.4%^26^ for women. This discrepancy is due in part to the method used to measure body fat percentage. The current gold standard is dual-energy X-ray absorptiometry (DXA), which provides detailed analysis of body composition, including the distribution of body fat and lean mass^31^. While other methods are available, such as bioelectrical impedance analysis, skinfold measurements, and air displacement plethysmography, DXA offers higher precision and reliability in quantifying regional and global body composition. It is also much safer, more available and less expensive than advanced methods such as computed tomography or magnetic resonance imaging^32^. The rationale for incorporating DXA into clinical practice as an essential tool for identifying individuals at risk of metabolic disease was presented by De Lorenzo et al. in their recently published narrative review^28^. Given these methodological considerations and the growing importance of accurate body composition assessment, further research is needed to establish population-specific reference values and risk thresholds.

The present study aimed to analyse the body composition of young women (aged 18–24 years) with normal BMI, residing in north-western Poland, in order to assess the relationship between adipose tissue distribution and cardiometabolic risk. The primary objective was to evaluate the prognostic value of fat and fat-free indicators in predicting the NWO phenotype and its accompanying metabolic abnormalities. Additionally, we sought to establish region-specific reference values for body fat percentage thresholds in this population, using DXA measurements as our primary assessment method. We hypothesized that specific patterns of fat distribution, even within the normal BMI range, would be significantly associated with cardiometabolic risk factors.

## Materials and methods

The research was approved by the Bioethics Committee of the Pomeranian Medical University in Szczecin (no. KB-0012/13/2021). The research was carried out in the Diagnostic Laboratory of the Department of Functional Diagnostics and Physical Medicine of the Pomeranian Medical University in Szczecin in the period from June 2021 to December 2023.

### Recruitment strategy

Participants were recruited using convenience sampling. Recruitment was conducted through advertisements on a social networking site and through notice boards on the academic campus (where the 3 largest universities in the region have their departments). The advertisement included basic information: the purpose of the study, inclusion/exclusion criteria (presented in the next paragraph), and contact details for the principal investigator. A total of 412 candidates applied, of whom 330 met all the criteria and provided informed written consent to participate (a recruitment response rate of 80.1%).

### Volunteers

The study included young women aged 18–24 years with normal BMI (18.5–24.9 kg/m^2^), residing in the urban area in western Poland, from a university environment. Excluded from the study were women who smoked, had metal implants, were physically active (athletes or regular competitive exercisers), reported vitamin and/or mineral supplementation, reported acute and/or chronic illnesses, used hypolipemic, antihypertensive, antiglycemic or insulin medications, were pregnant or breastfeeding or on hormone therapy, were undergoing nutritional monitoring and/or had changed their usual diet in the past 6 months. The final study population comprised 330 women. Before starting the research, each volunteer read the information regarding the research and planned procedures and gave written consent to participate in the study (in accordance with the Declaration of Helsinki), to process personal data and to use the results for scientific purposes.

## Research procedures

The research procedures included 4 stages: venous blood collection, anthropometric measurements, body composition analysis and biochemical analysis. Based on the results obtained, an abnormal metabolic phenotype was diagnosed among the examined women.

Before starting the procedures, blood pressure and heart rate were measured using an Omron M6 Comfort blood pressure monitor (Omron Healthcare, Kyoto, Japan).

### Blood collection

Blood samples were collected after an overnight fast from 6:00 a.m. to 8:00 a.m., after 10 min of rest in a sitting position, by qualified medical personnel from the antecubital vein, in accordance with applicable standards, using a Vacutainer® vacuum suction device (Vacutainer System, BD- Plymouth, PL6 7BP, UK). 15 ml of blood was collected each time. To obtain plasma, test tubes containing EDTA-K_2_ were used. To obtain the serum, test tubes with a coagulation activator sprayed on them were used and, after collection, they were stored for about 30 min at room temperature until a clot was obtained. Then the blood was centrifuged (3000 rpm; 15 min; 4 °C) using an MPW-260R centrifuge from MPW Med. Instruments Poland. Centrifuged plasma and serum were aliquoted and stored at − 70 °C in the Eppendorf® CryoCube® F570 freezer until further analysis.

### Anthropometric measurements

Anthropometric measurements included height and weight, as well as waist and hip circumferences. During the measurements, the participants wore minimal clothing. Body height and weight were measured using a Seca stadiometer with an accuracy of 0.01 kg and 1 mm (Seca 711). Waist circumference (WC) was measured halfway between the lower edge of the rib and the iliac process. Hip circumference (HC) was measured above the buttocks at the widest point around the greater trochanter. Circumferences were measured using an anthropometric tape with an accuracy of 1 mm (Seca 203). Each time, the measurements were performed twice, and the arithmetic mean of the obtained results was used for further analyses. Based on the obtained results, body mass index (BMI), waist to hip ratio (WHR) and waist to height ratio (WHtR) were calculated based on commonly used formulas:

$$BMI=\frac{weight \;(kg)}{{height}^{2}\;(m)}$$

$$WHR=\frac{waist\; {circumference }\;(\text{cm})}{hip \;{circumference }\;(\text{cm})}$$

$$WHtR= \frac{waist\; circumference \;(cm)}{{height}^{2} \;(m)}$$

### Body composition analysis

The body composition of the volunteers was measured by dual energy X-ray absorptiometry (DXA) using the Hologic device, QDR 4500W®, (Quirugil, Bogota, Colombia) with Hologic APEX software (APEX Version 5.6.0.4, Hologic Inc., Marlborough, MA, USA; https://www.hologic.com) performing a high-resolution whole-body scan. According to the manufacturer, the device is characterized by the following values of measurement repeatability and precision: 1) intraclass correlation coefficients for total fat mass, percentage of body fat and lean soft tissue: 0.997–0.999; 2) coefficient of variation for fat mass: 1.2%; for lean mass: 1.1% In order to ensure reliability and high quality of the results, the device was calibrated in accordance with the manufacturer’s recommendations (daily using a spine phantom).

The participants were positioned according to the manufacturer’s recommendations—on their backs, arms along the body, thumb on the iliac crest, remaining fingers spread, legs internally rotated by 25 degrees. During the examination, the patient lay still and breathed normally.

The Hologic APEX software adjusts the gynoid and android regions and visceral fat tissue (VAT) based on the patient positioning described above. The operator’s task is to check the correctness of the adjustment of the regions: android—20% of the distance from the horizontal line of the pelvis to the neck line, the lower border coincides with the horizontal line of the pelvis, the side borders coincide with the lines of the shoulders; gynoid—height twice as high as android region, upper border below the horizontal line of the pelvis by a distance equal to 1.5 times the height of the android region, lateral borders coincident with the lines of the shoulders. In order to adjust the VAT areas, it is necessary to verify the correctness of marking of the abdominal region, the edge of the subcutaneous fat and the visceral cavity. To eliminate human error and ensure reliable results, scans were performed and analyzed by a single, trained technician.

Fat and fat-free indicators were used for further analysis. Fat indicators included: percent body fat (PBF); fat mass (FM); fat mass index (FMI; FM/Height^2^); appendicular fat mass (AFM); appendicular fat mass ratio (AFMI; AFM/Height^2^); trunk-to-appendicular fat ratio (TAR); ratio between the percent of the trunk fat mass and the percent of the lower-limb fat mass (fat mass ratio; FMR); android percent fat (APF), gynoid percent fat (GPF), android to gynoid ratio (A/G), visceral fat area (VFA), visceral fat mass (VFM) and visceral fat volume (VFV). Fat-free indicators included: fat free mass (FFM—sum of TLM and bone mineral content), fat free mass index (FFMI; FFM/Height^2^), appendicular fat-free mass (AFFM), appendicular fat-free mass index (AFFMI; AFFM/Height^2^), total lean mass (TLM—includes the mass of muscles, internal organs and connective tissue, but does not include the mineral content of bones), total lean mass index (TLMI; TLM/Height^2^), appendicular lean mass (ALM) and appendicular lean mass index (ALMI; ALM/Height^2^).

### Biochemical analysis

A spectrophotometric method was used to determine the concentration of total cholesterol (TC), HDL cholesterol (HDL-C), triglycerides (TG), glucose and high-sensitivity C-reactive protein (hs-CRP). The concentration of LDL cholesterol fraction (LDL-C) was determined using the direct method. The analyses were performed in the accredited Medical Diagnostic Laboratory of the Laboratory Medicine Team of the 109th Military Specialist Hospital of the Independent Public Health Care Facility with an Outpatient Clinic in Szczecin.

Non-high-density lipoprotein cholesterol (non-HDL-C) was calculated using the formula: TC – HDL-C. After the determinations, the obtained lipid profile was supplemented with the calculation of the TG/HDL ratio. Based on the results of glucose and triglyceride concentrations, the triglycerides–glucose index (TG/G) was calculated according to the formula: natural logarithm[fasting triglycerides (mM) × fasting glucose (mM)/2]^33^). Insulin concentrations were determined using commercially available enzyme-linked immunosorbent assay (ELISA) kits (Demeditec Diagnostics GmbH, Kiel, Germany). The assay range was between 1.76 and 100 μlU/mL, intra-assay: < 2.2% and inter-assay: < 4.4%. Based on the results of insulin and glucose concentrations, the Homeostatic Model Assessment for Insulin Resistance (HOMA-IR) index was calculated according to the formula: fasting plasma insulin concentration (mU/L) × fasting plasma glucose concentration (mM)/22.5).

Based on the anthropometric and biochemical results obtained, the following indices were calculated:

1. Visceral adiposity index (VAI), according to the formula^34^:
  $$\left(\frac{\text{WC }(\text{cm})}{36.58+\left(1.89 \times \text{ BMI }(\frac{\text{kg}}{{m}^{2}})\right)}\right)\times \left(\frac{\text{TG }(\text{mM})}{0.81}\right)\times \left(\frac{1.52}{\text{HDL}-\text{C }(\text{mM})}\right)$$
2. Lipid accumulation product (LAP), according to the formula^35^:
  $$\text{TG }(\text{mM}) \times (\text{WC }(\text{cm})-58)$$
3. Cardiometabolic index (CMI), according to the formula^36^:
  $$\frac{\text{TG }(\text{mM}) }{\text{HDL}-\text{C }(\text{mM}) } \times \text{ WHtR}$$

## Statistical analysis

The minimal size of the study sample was estimated using the G*Power software (Version 3.1.9.4, https://www.psychologie.hhu.de/arbeitsgruppen/allgemeine-psychologie-und-arbeitspsychologie/gpower). The effect size was set at 0.5, the error probability α was 0.05, and the power was 0.95. The non-central parameter δ was 3.317. The total sample size was 176 and the actual power was 0.951.

In order to enhance the precision and robustness of our findings, we elected to enroll 330 women rather than the minimum 176. This larger sample size was chosen to (1) achieve narrower confidence intervals around key estimates, (2) allow for potential attrition or missing data, (3) support adequately powered subgroup analyses (normal weight vs. normal weight obesity), and (4) ensure sufficient sample size for multivariable modeling that adjusts for confounders. Our funding and resource availability permitted this expansion, thereby increasing the credibility and generalizability of our results.

Statistical analysis of the obtained results was performed using Statistica 13.3 software (Statistica PL, StatSoft, Kraków, Poland). The Shapiro–Wilk test was used to check the normality of the distribution. The distribution of the data deviated from normal, therefore non-parametric tests were used for detailed statistical analyses. The results were presented as median and interquartile range 25% to 75% (Q25–Q75). The Mann–Whitney U test was used to demonstrate the significance of differences. Spearman correlation coefficients were used to describe associations between continuous variables; their values are presented along with 95% confidence intervals. The level of significance was set at *p* < 0.05.

Because multiple comparisons were performed simultaneously (multiple indicator analyses), the Holm–Bonferroni correction was used to control type I error. For all significance tests performed, adjusted p levels were calculated using the Holm procedure, and both raw and adjusted p (Holm) values are reported in the text and tables.

The predictive value of individual indicators of body fat content and distribution for predicting an unhealthy metabolic profile was assessed using ROC curves. The significance of a parameter was assessed using the area under the curve (AUC). Sensitivity, specificity, Youden index and cut-off value were calculated for each indicator.

## Results

### Diagnosis of the metabolically abnormal phenotype

Considering six cardiometabolic abnormalities, using the definition recommended by Wildman et al.^37^ (Table 1), we classified participants as metabolically unhealthy if they met one or more of the cardiometabolic abnormalities listed in Table 1 and metabolically healthy if they did not meet any cardiometabolic abnormality.

**Table 1:** Cardiometabolic biomarkers in the diagnosis of the metabolically abnormal phenotype in women.

Cardiometabolic abnormalites	
1. Elevated BP: systolic/diastolic BP ≥ 130/85 mm Hg or antihypertensive medication use	
2. Elevated TG: fasting TG ≥ 1.7 mM	
3. Decreased HDL-C level: HDL-C level < 1.3 mM in women or lipid-lowering medication use	
4. Elevated glucose level: FBG ≥ 5.6 mM or antidiabetic medication use	
5. Insulin resistance: HOMA-IR > 4.65 (i.e., the 90th percentile)	
6. Systemic inflammation: hs-CRP level > 2.2 mg/dl (i.e., the 90th percentile)	

*BP* blood pressure, *TG* triglycerides, *HDL-C* high-density lipoprotein cholesterol, *FBG* fasting blood glucose, *HOMA-IR* homeostatic model for insulin resistance, *hs-CRP* high-sensitivity C-reactive protein.

ROC curve analysis showed that PBF had the largest area under the curve (AUC) of 0.895 (95% CI 0.837–0.954). The optimal cut-off value for PBF in women was 35.78 based on a Youden index of 0.71 (sensitivity = 0.949, specificity = 0.763). However, the percentage of android tissue had a Youden index of 0.67. CI had the best specificity of 0.848 and PBF had the best sensitivity. The results of the ROC curves generated are shown in Table 2.

**Table 2:** Comparison of body composition indices in predicting the metabolically unhealthy phenotype among normal-weight women.

	AUROC	Sensitivity (%)	Specificity (%)	Younden index	Cutoff	P value	95%CI
TFM	0.833	61.6	90.2	0.60	8.65	0.001	0.759–0.906
PBF	0.895	94.9	76.3	0.71	35.78	0.001	0.837–0.954
AF (%)	0.877	82.1	84.8	0.67	34.43	0.001	0.810–0.944
GF (%)	0.848	92.3	73.9	0.66	40.98	0.001	0.779–0.918
A/G ratio	0.776	87.2	66.3	0.53	0.77	0.001	0.693–0.859
FMR	0.679	89.7	40.2	0.30	0.79	0.001	0.583–0.774
FFM	0.436	0.00	0.98	0.02	31.2	0.238	0.329–0.543
TLM	0.433	0.05	0.92	0.02	33.3	0.221	0.327–0.540
WHR	0.531	0.44	0.73	0.17	0.75	0.572	0.423–0.640
WHtR	0.734	0.58	0.77	0.36	43.22	0.001	0.638–0.829

*AUROC* area under the receiver operating characteristic curve, *CI* confidence interval, *TFM* total fat mass, *PBF* percent body fat, *AF* Android fat, *GF* Gynoid fat, *A/G ratio* Android fat to Gynoit fat, *FMR* fat mass ratio, *FFM* fat-free mass, *TLM* total lean mass, *WHR* waist to hip ratio, *WHtR* waist to height ratio.

Table 2 Comparison of body composition indices in predicting the metabolically unhealthy phenotype among normal-weight women.

The diagnostic ability of PBF was the best, therefore, based on the determined cut-off point (35.78% BF), women with BMI < 25 kg/m^2^ were divided into two groups: NW (normal weight; BMI 18.5–24.9 kg/m^2^, PBF < 35.78%) and NWO (normal weight obesity; BMI 18.5–24.9 kg/m^2^, PBF > 35.78%). Finally, 90 women were in the NWO group, representing 27.3% of the subjects, among whom 55.7% had one or more of the cardiometabolic abnormalities listed in Table 1. The NW group included 11.2% of women with an abnormal metabolic phenotype.

The median age is identical in both groups (20 years). Women in the NWO group had significantly (*p* = 0.001) higher body weight (62 vs. 58 kg) and larger WC (73 vs. 69 cm) and HC (99 vs. 94 cm). Also BMI and WHtR values were significantly (*p* = 0.001) higher among NWO women (BMI: 22.8 vs. 20.7 kg/m^2^; WHtR: 43 vs. 41). Detailed values of the analyzed anthropometric parameters are presented in Table 3.

**Table 3:** Anthropometric characteristics of the subjects.

	All		NW		NWO		*P*-value	Corrected *P*-value
	Median	Q_25_–Q_75_	Median	Q_25_–Q_75_	Median	Q_25_–Q_75_		
Age (years)	20	19–22	20	19–22	20	19–22	0.615	1
Weight (kg)	61	55–66	58	54–63	62	57–67	0.000*	0.001
Height (cm)	167	163–172	169	163–174	166	162–172	0.073	0.220
BMI (kg/m^2^)	21.6	20.31–23.20	20.7	19.59–22.19	22.8	21.36–23.81	0.000*	0.000*
WC (cm)	70	67–74	69	66–73	73	69–75	0.000*	0.002
HC (cm)	96	91–100	94	90–97	99	95–103	0.000*	0.000*
WHR	0.7	0.70–0.76	0.7	0.71–0.76	0.7	0.70–0.76	0.621	1
WHtR	42	40–44	41	39–42	43	41–45	0.000*	0.000*

*BMI* body mass index, *WC* waist circumference, *HC* hip circumference, *WHR* waist to hip ratio, *WHtR* waist to height ratio; * *p* < 0.0001.

Body composition analysis showed that NWO women were characterized by significantly (*p* = 0.001) higher fat deposit content, both in relative (PBF, FM, AFM) and absolute (FMI, AFMI) indices. No statistically significant differences were found in TAR values (*p* = 0.068), while FMR was significantly higher in NWO women (*p* = 0.001). The android to gynoid fat ratio (A/G) was significantly higher in NWO women (*p* = 0.001). Analysis of visceral adipose tissue deposition (VFA, VFM, VFV) showed significantly higher values in the NWO group (*p* = 0.001). The analysis of lean body mass showed significantly (*p* = 0.001) lower values of both relative indices (FFM, AFFM, TLM, ALM) and absolute indices (FFMI, AFFMI, TLMI, ALMI). Detailed values of body composition analysis are presented in Table 4.

**Table 4:** Body composition characteristics of the subjects.

	All		NW		NWO		*P*-value	Corrected *P*-value
	Median	Q_25_–Q_75_	Median	Q_25_–Q_75_	Median	Q_25_–Q_75_		
PBF (%)	35.2	32.09–38.43	32	29.49–33.35	38.1	36.58–39.73	0.000*	0.000*
FM (kg)	21.8	19.01–24.93	18.5	16.65–20.87	24.7	22.56–26.45	0.000*	0.000*
FMI (kg/m^2^)	7.8	6.74–8.95	6.7	6.1–7.12	8.8	8.35–9.54	0.000*	0.000*
AFM (kg)	12	10.36–13.65	10.4	9.17–11.41	13.4	12.2–14.52	0.000*	0.000*
AFMI (kg/m^2^)	4.3	3.7–4.86	3.7	3.35–3.97	4.8	4.43–5.13	0.000*	0.000*
TAR	0.7	0.66–0.79	0.7	0.65–0.78	0.7	0.68–0.81	0.068	0.203
FMR	0.7	0.69–0.79	0.7	0.67–0.76	0.8	0.71–0.8	0.000*	0.003
APF (%)	31.4	27.9–35.79	27.9	23.85–30.15	35.6	32.71–38.11	0.000*	0.000*
GPF (%)	40.7	38.1–43.02	37.7	35.1–39.35	42.9	41.57–44.76	0.000*	0.000*
A/G	0.8	0.71–0.84	0.7	0.67–0.8	0.8	0.76–0.89	0.000*	0.000*
VFA (cm^2^)	45.7	36.16–57.17	40	33.68–48.76	52	42.26–64.85	0.000*	0.000*
VFM (g)	220.4	174.34–275.64	192.7	162.37–235.11	250.6	203.77–312.67	0.000*	0.000*
VFV (cm^3^)	238.2	188.48–297.99	208.3	175.54–254.17	271	220.29–338.02	0.000*	0.000*
FFM (kg)	37.9	35.89–41.16	38.9	36.38–41.88	37.4	35.49–40.55	0.041	0.188
FFMI (kg/m^2^)	13.7	13.02–14.4	13.9	12.96–14.42	13.6	13.05–14.28	0.297	0.297
AFFM (kg)	16.3	14.91–17.71	16.7	15.5–18.43	16	14.72–17.51	0.015	0.122
AFFMI (kg/m^2^)	5.8	5.5–6.22	5.9	5.59–6.39	5.8	5.45–6.07	0.000*	0.000*
TLM (kg)	40.3	37.99–43.39	41	38.54–44.3	39.5	37.53–42.91	0.035	0.203
TLMI (kg/m^2^)	14.6	13.79–15.24	14.7	13.8–15.3	14.3	13.79–15.13	0.022	0.155
ALM (kg)	17.5	15.98–18.87	17.9	16.52–19.54	17	15.75–18.66	0.014	0.188
ALMI (kg/m^2^)	6.2	5.91–6.6	6.3	5.97–6.84	6.2	5.81–6.49	0.028	0.169

*PBF* percent body fat, *FM* fat mass, *FMI* fat mass index, *AFM* appendicular fat mass, *AFMI* appendicular fat mass ratio, *TAR* trunk-to-appendicular fat ratio, *FMR* fat mass ratio, *APF* android percent fat, *GPF* gynoid percent fat, *A/G* android percent fat to gynoid percent fat ratio, *VFA* visceral fat area, *VFM* visceral fat mass, *VFV* visceral fat volume, *FFM* fat-free mass, *FFMI* fat-free mass index, *AFFM* appendicular fat-free mass, *AFFMI* appendicular fat-free mass index, *TLM* total lean mass, *TLMI* total lean mass index, *ALM* appendicular lean mass, *ALMI* appendicular lean mass index; * p < 0.0001.

Analysis of basic hemodynamic parameters (HR, SBP, DBP) showed significantly higher HR values in the NWO group (84 vs 79 bpm; *p* = 0.01). SBP and DBP values were also higher (SBP: 113 vs 109 mmHg; DBP: 76 vs 74 mmHg), but these were not statistically significant differences (Supplementary material Figure S1.).

Women with NWO were characterized by significantly higher glucose (4.4 vs 3.9 mM; *p* = 0.03) and insulin (13.4 vs 10.4 U/ml; *p* = 0.003) levels compared to the NW group. HOMA-IR value was also significantly higher (2.3 vs 1.7; *p* = 0.002) (Supplementary material Figure S2.).

Lipid profile analysis showed that NWO women were characterized by higher levels of TC (3.4 vs 2.8 mM; *p* = 0.02), LDL-C (2.1 vs 1.6 mM; *p* = 0.01), non-HDL (2.3 vs 1.7 mM; *p* = 0.01) and TG (0.8 vs 0.6 mM; *p* = 0.01) compared to the NW group. HDL-C values were lower in the NWO group (1.9 vs 1.2 mM; *p* = 0.05) (Supplementary material Figure S3.).

NWO women were characterized by higher values of all metabolic parameters, with TG/G (3.4 vs 3.3) and VAI (10.3 vs 6) being significantly higher (TG/G *p* = 0.02; LAP *p* = 0.001). In the case of TG/HDL-C (0.6 vs 0.5), VAI (1 vs 0.9) and CMI (25 vs 22.2), the differences were not statistically significant (Supplementary material Figure S4.). Comparisons of hemodynamic, metabolic and lipid parameters between NW and NWO groups are presented in the supplementary material (Table S1).

In the analysis of the relationship between body composition, including the content and distribution of fat tissue, and the assessed metabolic profile, we demonstrated relationships between fat distribution variables and insulin sensitivity variables (shown in Table 5). The percentage of body fat correlated positively with HOMA-IR value (r = 0.18; 95% CI [0.07–0.28]; *p* < 0.05) and plasma insulin level (r = 0.22; 95% CI [0.11–0.32]; *p* < 0.02). The android to gynoid fat ratio was positively correlated with HOMA-IR value (r = 0.21; 95% CI [0.10–0.31]; *p* < 0.05) and plasma insulin level (r = 0.23; 95% CI [0.12–0.33]; *p* < 0.02). The percentage of android fat was also significantly and positively correlated with fasting insulin concentration (r = 0.22; 95% CI [0.11–0.32]; *p* < 0.02). None of the fat distribution variables had significant correlation with fasting glucose concentration.

**Table 5:** Correlation coefficients and 95% confidence intervals for association between fat distribution variables and markers of insulin resistance.

	HOMA-IR	Plasma glucose level	Plasma insulin level
PBF (%)	0.18^a^ (0.07–0.28)	0.05 (− 0.06–0.16)	0.22^b^ (0.11–0.32)
APF (%)	0.17 (0.06–0.27)	0.02 (− 0.09–0.13)	0.22^b^ (0.11–0.32)
GPF (%)	0.08 (− 0.03–0.19)	0.008 (− 0.10–0.12)	0.10 (− 0.01–0.21)
A/G	0.21^a^ (0.10–0.31)	0.03 (− 0.08–0.14)	0.23^b^ (0.12–0.33)

*PBF* percent body fat, *APF* android percent fat, *GPF* gynoid percent fat, *A/G* android percent fat to gynoid percent fat ratio; ^a^* p* < 0.05; ^b^* p* < 0.02.

In our studies, we also demonstrated a significant, negative relationship between HDL-C concentration and A/G ratio (r = − 0.32; 95% CI [− 0.41 to − 0.22]; *p* = 0.001). None of the anthropometric indicators of obesity (BMI, WC, WHR, WHtR) showed a significant relationship with blood pressure, lipid profile parameters and insulin resistance indicators.

## Discussion

### Main findings of the study

Our study provides evidence for the occurrence of adverse metabolic changes in young, apparently healthy, normal-weight women (NWO). Key findings include: (1) identification of the optimal PBF cut-off point at 35.78% as a potential target for the diagnosis of cardiometabolic disorders, (2) a high prevalence of the NWO phenotype (27.3%) in the studied population of young women, (3) significant associations between adipose tissue distribution and metabolic parameters, with particular emphasis on the role of the A/G ratio, and (4) clear differences in lipid profile and insulin sensitivity between the NW and NWO groups.

### The Importance of body fat measurement and PBF cut-off point

The young and non-obese women we studied were selected from the local population, university environment. Based on the established cut-off point for PBF, we divided them into two groups (NW and NWO). This classification and subsequent analyses allowed us to make several observations on the relationship between body composition, including the content and distribution of adipose tissue, and insulin resistance and plasma lipid profile. First, BMI cannot be used as a commonly used criterion to determine whether a given person has a metabolic profile associated with increased cardiometabolic risk or not. Apparently, “lean” people can have an atherogenic lipid profile and can be insulin resistant. Second, the A/G ratio is positively correlated with insulin resistance, and negatively with HDL-C concentration. This relationship is independent of BMI, WC, WHR and WHtR. Thus, in women with NWO, the accumulation of adipose tissue in the upper body is associated with a higher HOMA-IR index and a lower HDL-C concentration. Third, in women with NWO, in addition to a lower HDL-C concentration, a further change in the lipoprotein profile is observed, in particular an increase in TG, LDL-C and non-HDL-C concentrations, which additionally increases the risk of atherogenicity and cardiovascular disease^38,39^.

Obesity is defined as an excess of adipose tissue, the size of which correlates with the development of many comorbidities^40^. A growing number of studies indicate that the amount of body fat plays a key role in increasing cardiometabolic risk^41–43^. Despite this, the quantitative determination of body fat is still little used in the risk assessment of e.g., cardiometabolic diseases also in people with a normal BMI. Therefore, in this study, taking into account six cardiometabolic abnormalities^37^ we examined the associations between indicators related to body fat content and its distribution an unhealthy metabolic profile. The diagnostic ability of PBF was the best, therefore, based on the designated cut-off point of 35.78% of PBF, we divided women with BMI < 25 kg/m^2^ into two groups: NW (normal weight; BMI 18.5–24.9 kg/m^2^, PBF < 35.78%) and NWO (normal weight obesity; BMI 18.5–24.9 kg/m^2^, PBF > 35.78%). Determining the optimal PBF cut-off point is crucial for the early identification of individuals at increased cardiometabolic risk, particularly among young adults with normal body weight.

Several different cut-off points for PBF have been proposed in the literature. Kim et al.^44^ used a PBF threshold of ≥ 20.6% for Korean men and ≥ 33.4% for women to define NWO, whereas Romero-Corral et al.^15^ classified 6171 Americans as NWO according to a PBF of > 23.1% in men and > 33.3% in women. In another study of 1222 Brazilians aged 23–25 years, NWO was defined when PBF exceeded > 23% in men and > 30% in women^45^. Similar thresholds were adopted in the studies of Jia et al. (≥ 24% for men and ≥ 33% for women)^46^, Liu et al. (> 20% for men and > 30% for women)^47^, Hariyanto et al. (> 25% for men and > 30% for women)^48^, Xu et al.^49^, Wang et al.^50^ (> 25% for men and > 35% for women)^51^, Ferjančeková et al. (> 30% for women), Wang et al. (> 33.3% for women)^52^, Kim et al. (≥ 26% for men and ≥ 36% for women)^53^, Yogesh et al.^54^, Aruna et al.^55^ (≥ 20.6% for men and ≥ 33.4% for women), Santos et al.^56^ and Passos et al. (> 19% for men and > 30% for women)^57^. PBF values above ≥ 25% for men and ≥ 35% for women were used to assess the combined effect of BMI and PBF on the prognosis of coronary heart disease^58^, while in the prediction of obesity-related cardiovascular risk factors, except for dyslipidemia, the optimal PBF limit was found to be 25.8% for men and 37.1% for women^59^. In studies among Chinese students^60^ and Swedish adults (45–75 years old)^22^, NWO was classified at PBF ≥ 20% for men and ≥ 30 for women. Olafsdottir et al., studying 18-year-old Icelandic subjects, adopted thresholds of > 17.6% for men and > 31.6% for women, respectively^61^. In turn, Ma et al. defined NWO as the highest tertile of PBF^62^. Therefore, there are no clearly defined cut-off values for PBF, which may be due to the lack of uniform diagnostic criteria for NWO, the measurement methods used (DXA, BIA), or the population studied. Age, gender, ethnicity/race may affect the distribution of body fat and body composition. It therefore seems that the proposed PBF thresholds are only applicable to the groups for which they were estimated or to groups similar in terms of age, gender, and ethnicity. Despite the differences in the presented results, it can be seen that the cut-off threshold for men is about 10% lower than for women and that the cut-off values for PBF indicate a difference of ± 5% around the threshold of 25% for men and 35% for women, which is the one we determined in our study and the one used to define NWO in several other studies^19,63–65^.

### Prevalence of the NWO phenotype

The prevalence of the NWO phenotype in our population was 27.3%. This means that approximately one in four “lean” women aged 18–24 years exhibits the NWO phenotype. These findings indicate a high prevalence of NWO in early adulthood. Correa-Rodríguez et al.^18^ showed that the prevalence of NWO in Latin American women, defined by a normal BMI (18.5–24.9 kg/m^2^) and PBF ≥ 38.9%, was 46%, whereas in Korean women aged 20 years and older (PBF ≥ 36%) it was 25%^66^. In contrast, in another study, Romero-Corral et al.^15^ found that the prevalence of the NWO phenotype among American women was 33.4% (PBF ≥ 33.3%), while Peterson et al.^67^ reported that almost half of the women with normal weight (BMI < 25 kg/m^2^) were actually obese according to PBF (≥ 35%). These results differ from those of Madeira et al.^45^, who reported that the prevalence of NWO among young Brazilians aged 23–25 years was 9.1%. It should be added, however, that in this study, body fat content was assessed by measuring the sum of subscapular and triceps skinfolds above the sex-specific 90th percentiles of the study sample. Similarly, using the highest tertile of body fat percentage as the cut-off value, in a study conducted among adults aged 35–75 years in Switzerland, the prevalence of NWO was 5.4% in women^21^. In turn, studies among Chinese students showed that 25.5% of men and 40.1% of women were classified as NWO phenotype^60^. The percentage of adult Swedes (45–75 years old) with NWO was 22.2%^22^. In a cross-sectional study among Indonesians students (18–24 years old), 31.5% of the volunteers examined met the NWO criteria^48^. It seems, therefore, that the discrepancies in the data on the incidence of NWO may be due to the lack of consensus regarding the diagnostic criteria^21^, participant characteristics (age, lifestyle, dietary habits) and ethnic differences in the cohorts included in the study.

### Characteristics of body composition and its relationship with metabolic parameters

In our study, body composition variables of all participants, including the content and distribution of adipose tissue, were measured using dual X-ray absorptiometry (DXA). We observed that NWO women compared to NW, despite a normal BMI (18.5–24.9 kg/m^2^), showed a cluster of metabolically risky body composition features. This concerns in particular a significantly larger android adipose tissue deposition (higher APF), especially with the accumulation of visceral adipose tissue (higher VFM) and a higher percentage of android to gynoid fat (higher A/G ratio). Furthermore, our results showed that the same group of NWO women showed significantly higher insulin and HOMA-IR levels compared to NW and significant changes in the basal lipid profile towards its atherogenicity (significant increase in TC, LDL-C, non-HDL-C, TG and decrease in HDL-C).

### Lipid profile and insulin sensitivity in the context of NWO

Like us, several studies have associated NWO with an increased risk of dyslipidemia^15,21,30^. Kang et al.^30^, in a study of 164 Koreans, showed that individuals with NWO had higher TG levels and lower HDL-C levels compared with NW individuals. In Brazil, Madeira et al.^45^ observed an association between current low HDL-C levels (OR = 1.65; 95% CI, 1.11–2.47) and hypertriglyceridemia (OR = 1.93; 95% CI, 1.02–3.64). In a study of 6171 American adults of both sexes, Romero-Corral et al.^15^ reported that individuals with NWO had lower serum HDL-C levels, higher LDL-C and TG levels, and altered apolipoprotein B to apolipoprotein A1 ratios compared with individuals with normal BMI and PBF. They also observed a proportional association between increased PBF and the highest risk of dyslipidemia and cardiovascular mortality among women with NWO (HR = 1.06 for each point increase in percent body fat; 95% CI, 1.01–1.12). Studies conducted in Iceland showed that the NWO group was 2.2 times more likely to exhibit one or more risk factors for metabolic syndrome (OR = 2.2; 95% CI 1.2–3.9) compared to the control group^61^. On the other hand, De Lorenzo et al.^68^ found no differences between the lipid profiles of Italian adults with NWO and control groups, although they found a higher LDL-C to HDL-C ratio. However, in this study, the NWO group had a small number of subjects (n = 28). Studies conducted in the Slovak population showed that the NWO group was characterized by higher TG values and lower HDL-C, LDL-C compared to the control group, but these differences were not statistically significant (*p* > 0.05)^49^. In turn, Ma et al. observed lower values of both HDL-C, LDL-C and TG in the NWO group than in the control group^62^. In the study by Aruna et al., the NWO group was characterized by significantly higher values of glucose, insulin and HOMA-IR concentrations compared to the control group^55^. The observed metabolic abnormalities in young women with NWO may indicate an early stage of development of cardiometabolic disorders, which emphasizes the importance of early identification of this phenotype.

### The importance of body fat distribution

Although our results are consistent with some previous findings, we extend them by providing evidence that not only higher PBF content but also adipose tissue distribution calculated as the A/G fat distribution index may be associated with several markers of cardiometabolic complications in the NWO phenotype. In our study, a positive, significant correlation was found between HOMA-IR and PBF (r = 0.18; 95% CI [0.07–0.28]; *p* < 0.05) and A/G ratio (r = 0.21; 95% CI [0.10–0.31]; *p* < 0.05). The analysis of HOMA-IR components showed that glucose itself did not show significant relationships, while insulin correlated with PBF, APF (r = 0.22; 95% CI [0.11–0.32]; *p* < 0.02) and A/G ratio (r = 0.23; 95% CI [0.12–0.33]; *p* < 0.02). In addition, a negative correlation was found between the A/G ratio and HDL-C concentration (r = − 0.32; 95% CI [− 0.41 to − 0.22]; *p* = 0.001). Although all correlations are statistically significant, their weak-to-moderate strength suggests caution—clinical decisions should not be based solely on these indicators. The 95% confidence intervals for these correlations, although relatively narrow, are close to zero, further emphasizing the need for cautious interpretation in a clinical context. Further prospective studies should clarify the prognostic significance of these associations.

The key mechanism mediating these associations is chronic, low-grade inflammation developing in adipose tissue, especially visceral. In contrast to subcutaneous tissue, visceral adipocytes and their resident macrophages demonstrate excessive activation of the NF-κB and JNK pathways, which results in increased synthesis of proinflammatory cytokines such as TNF-α, IL-6, and IL-1β, which travel to the liver via the portal vein, increasing gluconeogenesis and inhibiting insulin signaling via serine phosphorylation of IRS-1 in hepatocytes and skeletal muscle^69–71^. At the same time, along with adipocyte hypertrophy, local hypoxia induces stabilization of the HIF-1α factor, which ineffectively supports angiogenesis, and at the same time stimulates the expression of SMPD3—a key enzyme for the synthesis of ceramides, which then accumulate in the liver and muscles, directly blocking the insulin receptor signaling cascade^72,73^. Moreover, the secretion of pro-inflammatory adipokines such as resistin or visfatin by adipocytes additionally increases endoplasmic stress and the generation of reactive oxygen species in insulin-sensitive cells, which intensifies the dysfunction of insulin receptors and increases insulin resistance^74,75^.

In this study, young women with NWO had a significantly higher A/G ratio, which was associated with increased insulin resistance and significantly lower HDL-C concentration compared to NW women. Our cross-sectional data suggest that associations between body fat content and metabolic parameters may vary according to fat distribution patterns, though the temporal and causal relationships require further investigation. Android fat refers to the accumulation of adipose tissue around the trunk, while gynoid fat refers to the fat stored in the subcutaneous depot of the hips, thighs, and buttocks^76^. A higher A/G ratio is typically associated with an android pattern of fat storage, characterized by increased visceral fat (VFM) around the trunk. The A/G ratio is a pattern of adipose tissue distribution that is associated with an increased risk of metabolic syndrome in a community-based study of Chinese women^77^, in the elderly population^78^, and is an important predictor of the risk of metabolic and cardiovascular diseases in normal-weight children as well as in overweight and obese children^79^. It is also associated with incident T2DM^80^, insulin resistance in obese adolescents^81^, nonalcoholic fatty liver disease^82^. Similar to our study, Bantle et al.^83^ showed that the A/G ratio was positively correlated with HOMA-IR and Matsuda ISI in young adult women. However, this study focused on overweight and obese women, whereas our population included women of normal weight. Studies conducted in younger populations also showed that the A/G ratio was a measure of adiposity closely associated with both insulin resistance and dyslipidemia in children aged 7–17 years^79,81^. Our analysis complements these findings and extends them to the population of young adult women. The A/G ratio seems to be a particularly valuable marker of metabolic risk that can be used in clinical practice to identify individuals with normal body weight but increased cardiometabolic risk.

Another finding of this study is that NWO women have significantly higher levels of non-HDL-C, which reflects the total cholesterol content of atherogenic lipoproteins containing apolipoprotein B, including LDL, VLDL, intermediate-density lipoprotein, remnants, and lipoprotein(a)^84^. Higher levels of non-HDL-C are a prognostic factor for atherosclerosis, coronary events, and cardiovascular mortality^85^. It is also significantly associated with metabolic syndrome burden. Individuals with increased non-HDL-C levels are at approximately 42% greater risk of clustering metabolic abnormalities, which can further promote the likelihood of cardiovascular disease development in their lifetime^38^.

### Clinical significance

The results of our cross-sectional study may have important clinical implications, if validated in longitudinal studies. First, our preliminary findings suggest that body composition assessment could complement BMI in cardiometabolic risk evaluation. Longitudinal validation may provide evidence of the predictive value of body composition measures for cardiometabolic risk in young adults. Second, it is advisable to consider including DXA body composition testing in routine screening of young women, especially those with a family history of metabolic diseases. Furthermore, the studies suggest a need for early intervention in individuals with the NWO phenotype, even in the presence of normal body weight. These interventions may include lifestyle modification, with particular attention to physical activity and diet, which may affect adipose tissue distribution and metabolic parameters.

### Limitations

This study has several strengths. First, we used DXA with its advanced technology to measure body composition and body fat distribution. Second, the subjects were recruited from a well-defined population that represented a single ethnic group and was aged 18–24 years. The narrow age group should not be considered a limitation but a particular strength, as it eliminates the confounding effect of age and other age-related confounders that could have influenced the results of the analyses.

This study also has several limitations. The cross-sectional design is a limitation, as the data are correlational only and do not allow conclusions about cause and effect. Because of the observational nature of this study, we cannot rule out the possibility of confounding effects due to unmeasured confounders. Ethnic differences and the influence of racial/ethnic differences on the association between regional adipose tissue accumulation and cardiometabolic risk are also well known^86^. One should therefore be aware of the limitations of generalizing the results of this study to other groups and other countries. Furthermore, the lack of long-term follow-up prevents the assessment of the development of cardiometabolic disorders over time. Finally, our use of convenience sampling via campus notice boards and social-media may have introduced selection bias, as volunteers who respond to such calls often differ in socioeconomic status, health awareness and motivation from the general population. Despite an 80.1% response rate, the self-selection of participants limits the external validity of our findings beyond university-affiliated young women.

### Future research directions

Future studies should focus on: (1) validating the designated PBF cutoff point in other populations, (2) conducting prospective studies assessing the long-term consequences of the NWO phenotype, (3) assessing the effectiveness of different interventions in this group of participants, and (4) gaining a deeper understanding of the mechanisms linking adipose tissue distribution to metabolic disorders.

## Conclusion

Our study provides evidence of adverse metabolic changes in young women with the NWO phenotype. Of particular importance is the distribution of adipose tissue, expressed by the A/G ratio, which, similarly to PBF, is associated with metabolic parameters independently of BMI and other anthropometric indices. These results emphasize the need for a comprehensive assessment of body composition together with the assessment of the content and distribution of adipose tissue in identifying individuals at increased cardiometabolic risk, even at normal body weight. The innovative aspect of our approach is the simultaneous assessment of multiple adiposity indicators and their correlation with cardiometabolic risk factors, which may contribute to the development of more accurate screening tools for NWO in young women in clinical practice.

## Electronic supplementary material

Below is the link to the electronic supplementary material.

Supplementary Material 1

## Data Availability

The datasets used and/or analysed during the current study available from the corresponding author on reasonable request.
