# Supplementary material for: Diagnostic and prognostic value of adipose tissue content and distribution indicators for normal weight obesity in young women
Source: Sci Rep. 2025 Sep 30;15:33995. doi: 10.1038/s41598-025-12262-6 (PMC12485104; doi:10.1038/s41598-025-12262-6)
Supplement: Supplementary file 1 — Supplementary Material 1 [file 41598_2025_12262_MOESM1_ESM.docx]

**Figure S1. Comparison of heart rate and blood pressure in the NW and NWO groups.**

**Legend:** The values are expressed as median (Q25-Q75). HR – heart rate; SBP – systolic blood pressure; DBP - diastolic blood pressure; NW – normal weight; NWO – normal weight obesity.

**Figure S2. Comparison of insulin resistance markers in the NW and NWO groups.**

**Legend:** The values are expressed as median (Q25-Q75). HOMA-IR - homeostatic model assessment of insulin resistance; NW – normal weight; NWO – normal weight obesity.

**Figure S3. Comparison of lipid profile in the NW and NWO groups.**

**Legend:** The values are expressed as median (Q25-Q75). TC- total cholesterol; HDL-C - high density lipoprotein cholesterol; LDL-C - low density lipoprotein cholesterol; non-HDL- C (HDL-C subtracted by TC); TG – triglycerides; NW – normal weight; NWO – normal weight obesity.

**Figure S4. Comparison of calculated metabolic parameters in the NW and NWO groups**

**Legend:** The values are expressed as median (Q25-Q75). TG/HDL-C (serum levels of TG divided by HDL-C); TG/G - triglycerides– glucose index; VAI – visceral adiposity index; LAP - lipid accumulation product; CMI - cardiometabolic index; NW – normal weight; NWO – normal weight obesity.

**Table S1. Comparisons of hemodynamic, metabolic and lipid parameters between NW and NWO groups**

|  | **All** | | **NW** | | **NWO** | | P-value | Corrected P-value |
| --- | --- | --- | --- | --- | --- | --- | --- | --- |
|  | **median** | Q_25_–Q_75_ | **median** | Q_25_–Q_75_ | median | Q_25_–Q_75_ |  |  |
| HR [bpm] | 81 | 72-92 | 79 | 68-87 | 84 | 72-96 | 0.012 | 0.152 |
| SBP [mmHg] | 112 | 105-122 | 109 | 104-121 | 113 | 107-123 | 0.234 | 0.767 |
| DBP [mmHg] | 75 | 71-84 | 74 | 70-81 | 76 | 72-76 | 0.203 | 0.767 |
| Glucose [mM] | 4.1 | 3.30-4.90 | 3.9 | 3.10-4.65 | 4.4 | 4.01-5.10 | 0.030 | 0.211 |
| Insulin [μlU/ml] | 11.2 | 8.60-16.13 | 10.4 | 7.34-13.51 | 13.4 | 9.29-18.85 | 0.004 | 0.052 |
| HOMA-IR | 2.1 | 1.31-2.91 | 1.7 | 1.19-2.37 | 2.3 | 1.52-3.67 | 0.002 | 0.036 |
| TC [mM] | 3.2 | 2.60-4.30 | 2.8 | 2.40-4.10 | 3.4 | 2.75-4.40 | 0.019 | 0.187 |
| HDL-C [mM] | 1.2 | 0.93-1.54 | 1.9 | 0.89-1.53 | 1.2 | 1.03-1.55 | 0.037 | 0.223 |
| LDL-C [mM] | 1.8 | 1.40-2.50 | 1.6 | 1.25-2.303 | 2.1 | 1.50-2.65 | 0.013 | 0.161 |
| Non-HDL [mM] | 2.0 | 1.50-2.70 | 1.7 | 1.35-2.55 | 2.3 | 1.65-2.25 | 0.017 | 0.187 |
| TG [mM] | 0.6 | 0.50-0.95 | 0.6 | 0.50-0.85 | 0.8 | 0.60-1.00 | 0.019 | 0.187 |
| TG/HDL-C | 0.6 | 0.41-0.76 | 0.5 | 0.39-0.69 | 0.6 | 0.44-0.80 | 0.192 | 0.767 |
| TG/G | 3.3 | 3.15-3.55 | 3.3 | 3.07-3.49 | 3.4 | 3.19-3.57 | 0.026 | 0.206 |
| VAI | 1.0 | 0.68-1.35 | 0.9 | 0.65-1.19 | 1.00 | 0.73-1.37 | 0.271 | 0.767 |
| LAP | 7.7 | 5.00-13.00 | 6.0 | 4.50-10.00 | 10.3 | 6.80-15.45 | 0.000* | 0.003 |
| CMI | 23.6 | 17.14-33.56 | 22.2 | 16.42-28.32 | 25.0 | 18.08-35.00 | 0.134 | 0.671 |

**Legend:** HR – heart rate; SBP – systolic blood pressure; DBP – diastolic blood pressure; HOMA-IR – homeostatic model assessment for insulin resistance; TC – total cholesterol; HDL-C – high-density lipoprotein cholesterol; LDL-C – low-density lipoprotein cholesterol; Non-HDL – non-high-density lipoprotein cholesterol; TG – triglycerides; TG/HDL-C – triglycerides to HDL-C ratio; TG/G – triglycerides to glucose ratio; VAI – visceral adiposity index; LAP – lipid accumulation product;
CMI – cardiometabolic index. * p < 0.0001.
